# The duty of care and the right to be cared for: is there a duty to treat the unvaccinated?

**DOI:** 10.1007/s11019-023-10186-4

**Published:** 2024-01-05

**Authors:** Zohar Lederman, Shalom Corcos

**Affiliations:** 1 Department of Emergency Medicine, LKS Medical Faculty, University of Hong Kong, Hong Kong, Hong Kong; 2 Assuta Samson Hospital, Ashdod, Israel

**Keywords:** Duty of care, Right to be cared for, Supererogatory

## Abstract

Vaccine hesitancy or refusal has been one of the major obstacles to herd immunity against Covid-19 in high-income countries and one of the causes for the emergence of variants. The refusal of people who are eligible for vaccination to receive vaccination creates an ethical dilemma between the duty of healthcare professionals (HCPs) to care for patients and their right to be taken care of. This paper argues for an extended social contract between patients and society wherein vaccination against Covid-19 is conceived as essential for the protection of the right of healthcare providers to be taken care of. Thus, a duty of care is only valid when those who can receive vaccination actually receive it. Whenever that is not the case, the continuing functioning of HCPs can only be perceived as supererogatory and not obligatory.

## Introduction

Vaccine hesitancy has been one of the major obstacles to some sort of community-level immunity against Covid-19 in high-income countries, particularly before the emergence of the Omicron variant. It in fact may have had a major causal role in the creation of variants.^1^ Prior to the emergence of the Omicron variant, more than one million Israelis out of seven million eligible for vaccination refused to do so, constituting half of all severe cases (Sokol 2021). Only 65% of Americans eligible for vaccination have at the time the first draft of this paper was being written received at least one vaccination dose.^2^ Regardless of their reasoning, the unvaccinated risk themselves, their close ones, their local community and even people in other countries, as they increase the risk for the creation of variants (Jing et al. 2023). Most importantly for us here, they risk healthcare providers (HCPs), including emergency medicine physicians.

Seventy-five physicians in Florida, US, staged a walk-out to express their frustration with people who can but refuse being vaccinated against Covid-19 in 2021 (Barrabi 2021). Public hospitals in Israel have declared that they would no longer receive Covid patients, after the government reportedly reversed a promise to allocate more resources. The hospitals reasoned that—among other reasons—the unvaccinated exerted increasing burden on the capacity of hospitals to provide appropriate care (immediately thereafter a compromise was reached but some hospitals still refused to receive Covid patients).

Refusal by medical professionals and institutions to care for any patient violates the Hippocratic oath and the ethos of medicine, committed to care for all regardless of race, gender, political affiliation, ideology etc. (Arras 1988; Emanuel 1988). Such ethos is explained most often by referring to professional medical virtues, where the traditional virtuous physician is committed to care for her patients even at the price of her own well-being. Other than virtues, one common argument to support such ethos is that our medical studies are heavily subsidized (and for some the salaries are high) and thus we should be expected to reciprocate. Another oft-cited argument is that HCPs implicitly entered a negotiation or signed a social contract where society allows us to self-regulate and almost monopolize healthcare while we commit to offer care virtually at all times and under all circumstances (Arras 1988; Derse 2015; Huber and Wynia 2004; Daniels 1991; Reid 2005; Johnson and Butcher 2020; Malm et al. 2008; Upshur et al. 2005). This last argument will become particularly relevant below.

This paper does not discuss refusal of care by HCPs, even though such cases did occur during past pandemics where HCPs actually fled their infected cities (Fox 1988), or called in sick (Upshur et al. 2005). The paper rather asks whether HCPs—particularly emergency medicine practitioners—owea duty to care (occasionally referred to as a duty to care, duty to assist, or duty to treat) for the unvaccinated. They either do, or they do not, and in that case their willingness to provide care is supererogatory. For present purposes it may be granted that HCPs indeed have a special positive duty (Malm et al. 2008) of care because of the arguments mentioned. The main argument made here, however, is that such a duty is only valid when the right of HCPs to be cared for is fulfilled. That right to be cared for means that society and members of society ought to reduce the burden of HCPs and reciprocate them. In the present context, this responsibility entails protecting them from Covid, specifically vaccine compliance. The specific emphasis on vaccine compliance by patients as a condition to maintain a duty of care on the part of HCPs is what makes this paper novel.

Before discussing the duty of care in general, some clarificatory remarks are in order.

## Clarificatory remarks

First, the duties discussed therein are strictly ethical, not legal. While law should ideally rely on ethics, that is unfortunately not always the case (Derse 2015; Sokol 2012). For example, in Israel HCPs are legally permitted to force feed prisoners, even though force-feeding is generally considered unethical (Lederman and Lederman 2017). The opposite is logically false: ethical reasoning should not and cannot rely on laws or on what is common in legal practices. Inferring ethics from law is a kind of an is-ought gap and is considered to be a logical fallacy (Derse 1999).

Another clarificatory remark is that the paper focuses slightly more on emergency medicine providers and settings, for two main reasons. First, we are both emergency medicine physicians, and our initial concern and discussions about the issue at hand motivated this work in the first place. Second, the risk of asymptomatic transmission in the context of emergency medicine may be higher, as mentioned below. In any case, the arguments elaborated apply across the board.

A third clarificatory note is that vaccine hesitancy is understood as a delay in acceptance or refusal to receive vaccination despite availability of vaccines and appropriate supply and administration chains (WHO 2014; Bedford et al. 2018). The causes for and ways in which vaccine hesitancy occurs are complex, involving various individual, communal, societal, and governance factors. The philosophical enigma of free will and whether it is compatible with causal determinism also becomes pertinent if we seek to assign moral blame. At time of writing, both authors worked in a very specific healthcare context in which problems of access to primary healthcare including Covid vaccines are significantly less severe than in other places such as the US, but stigma and confidence in the medical and scientific expertise are still an issue. Any full account of moral responsibility would have to address such complexity, and others have indeed came close (Battin et al. 2009). Here, however, we conveniently restrict the discussion to cases in which individuals indeed had the free will (or just about enough of it) and the adequate access conditions to receive vaccination but still refused to do so. We at the same time acknowledge that both assumptions are controversial.

A fourth and last clarificatory remark relates to moral supererogation. This will prove useful over the next pages as we articulate and defend the claim that HCPs only have a morally supererogatory duty—rather than a moral duty—to care for the unvaccinated. What does this claim actually mean? Morally supererogatory acts are acts that go beyond the call of moral duty. They are good or praiseworthy but not required or obligatory from an ethical perspective; in other words, they are merely optional. Supererogatory acts are better, or are more praiseworthy, than acts that are not supererogatory. The agent, then, could have chosen to act in a suboptimal manner from a moral perspective—he would have been permitted to act, or justified in acting is such manner—but instead chose to act in an optimal manner. As seen in the case discussed therein, supererogatory acts often carry increased risk, burden, or costs to the individual—in fact, it is exactly the high costs or risks engendered by vaccine hesitancy that turn a duty of care into supererogatory. Costs, however, are not necessary for an act to be defined as supererogatory and they are not necessarily the reason for which an act is labeled as such. Rather, the source of supererogation is first the intended good motivating the act and second the voluntary nature of the act. Further, an act can be supererogatory whether it is acknowledged as such by the agent committing the act or not (Benn 2018; Benn and Bales 2020).^3^

Having outlined these four clarificatory remarks, we are now equipped to examine whether HCPs have a duty of care towards the unvaccinated. To anticipate, the conclusion is that HCPs merely have a supererogatory duty of care towards the unvaccinated. This means that HCPs should not be criticized for either refusing to treat unvaccinated patients or work in a healthcare system that does not ensure vaccination. And the fact that they continue caring for the unvaccinated or continue to work in such a system constitutes a supererogatory behavior even if is not acknowledged as such by the HCPs themselves.

## Duty of care

While originally considered a legal term (Kloss 1984; Sheahan and Lamont 2020), a duty of care is often cited to define the ethical obligation of HCPs to offer optimal treatment to their patients, based on their professional competency and relevant circumstances. A clinician is expected to discharge a duty of care even if it means bearing added risks (Emanuel 1988; Huber and Wynia 2004; Daniels 1991). The American Medical Association Code of Ethics for instance states that,

Because of their commitment to care for the sick and injured, individual physicians have an obligation to provide urgent medical care during disasters. This obligation holds even in the face of greater than usual risks to physicians’ own safety, health, or life. (AMACoMEO)

The American College of Emergency Physicians (ACEP) also establishes a duty of care: “…emergency physicians have a duty of justice to provide care to patients regardless of race, color, creed, gender, nationality, or other irrelevant properties” (American College of Emergency Medicine 2017).

In non-disaster settings, such duty of care is often presumed to exist without due argumentation (Malm et al. 2008); in other words, when they call on us, we go (Koven 2020).

Ethical dilemmas ensue when a clinician’s capability to provide adequate care is questioned (Emanuel 1988; Johnson and Butcher 2020; Lederman 2017; Holt 2008), or when the potential benefit to patients is minimal (Arras 1988; Emanuel 1988; Johnson and Butcher 2020), or when the risk to the HCP seems exceedingly high, as during terror attacks or natural disasters for example(Emanuel 1988; Daniels 1991; Johnson and Butcher 2020; Holt 2008). The AMA code further states that,

However, the physician workforce is not an unlimited resource. Therefore, when providing care in a disaster with its inherent dangers, physicians also have an obligation to evaluate the risks of providing care to individual patients versus the need to be available to provide care in the future. (AMACoMEO)

The ACEP also conditions a duty of care towards the individual patient on the common good, such that individual interests may be outweighed by societal considerations:

Though the emergency physician's duty to the patient is primary, it is not absolute. Emergency physician duties to the general public inform decision making on a daily basis; for example, the emergency physician has duties to allocate resources justly, oppose violence, and promote the public health that sometimes transcend duties to individual patients. (American College of Emergency Medicine 2017)^4^

Infectious disease (ID) outbreaks engendered perhaps the most controversy pertaining to a duty of care. Early writings seem to have considered a duty of care as a given (Huber and Wynia 2004). Contemporary authors however started wondering about the duty of care during HIV/AIDS, when HCPs had to face a minimally increased risk in caring for patients who were carriers of HIV or sick with AIDS. Because of such minimal extra risk, authors have generally upheld the duty of care and criticized HCPs for abandoning their patients, particularly if it stemmed from prejudice or unjust discrimination (Arras 1988; Emanuel 1988; Daniels 1991). As it became clear that ID outbreaks such as Severe Acute Respiratory Syndrome (SARS) (McDonald et al. 2004; Wong 2003) and Ebola Virus Disease (EVD) (Green 2014; McDiarmid and Crestani 2019) posed increased risks to HCPs—particularly those working in emergency departments—authors have again upheld a duty of care but emphasized societal obligations to reciprocate and protect HCPs (Reid 2005; McDiarmid and Crestani 2019).

One authoritative report on this matter stated that, “Health care providers will have to weigh demands of their professional roles against other competing obligations to their own health, and to family and friends” (Upshur et al. 2005, pp. 6–7). It also espoused the value of reciprocity, stating that, “Reciprocity requires that society support those who face a disproportionate burden in protecting the public good, and take steps to minimize burdens as much as possible” (Upshur et al. 2005, p. 7).

Writing about HCPs during SARS, the report portrays a picture that seems all too familiar:

“They faced an unknown and deadly communicable disease, a coronavirus for which there was no known effective treatment. They were rapidly forced to weigh serious and imminent health risks to themselves and their families against their duty to care for the sick.” Such description is obviously apt in the context of Covid as well, once we account for the much lower mortality rate of Covid compared to SARS which reached up to 10%.

A rare near-consensus was indeed born out of the rich deliberations on the topic: HCPs have a special positive duty of care towards their patients, which is quite distinct from a general positive duty of non-medical professionals to provide assistance to others when no significant or comparable (Singer 2011) risk is involved. A special positive duty means that society is justified in expecting HCPs to continue working despite of some degree of increased risk as part of their clinical practice (Malm et al. 2008). Society however has a corresponding or reciprocal duty or responsibility to mitigate that risk as much as possible, and to offer due compensation in case of harm. HCPs may also be justified in considering and protecting the interests of their loved ones, e.g. friends and family (Huber and Wynia 2004; Daniels 1991; Reid 2005; Johnson and Butcher 2020; McDiarmid and Crestani 2019). In other words, a duty of care applies *prima facie*, i.e. unless it is overridden by other considerations. Specifically, a duty of care applies only insofar it is reciprocated.

Authors, however, seem to have simply assumed that talk of a societal reciprocal obligation is straightforward. It is not. A society or community is eventually composed of individuals, collaborating to create a political, social etc. union that is conceptually and inspirationally greater than the sum of their own individual lives. ‘Societal obligations’ then mean both individuals’ behavior and relevant initiatives or policies implemented by the society as a whole, ideally ensured by a democratic governance. So who exactly holds a duty of reciprocity towards HCPs during pandemics such as Covid? And what does such a duty entail? The answer to the first question is that both individuals and society as a whole hold a duty or reciprocity towards HCPs, but holding individuals rather than society responsible is much more complex from a theoretical and pragmatic perspectives.

## Personal moral responsibility

Individuals act under complex psychological, political, philosophical and social influencing factors, and examining their behavior without due regard for these factors is philosophically unsavory, unjust, and impractical from a public health ethics perspective (Hansson 2022). Philosophers (Clark et al. 2015) have debated the plausibility and nature of personal moral responsibility in light of considerations of the compatibility of free will and causal determinism, and more specifically an agent’s ability to act otherwise (Frankfurt 1969), and moral luck (Frankfurt 1969; Williams 1982; Statman 1993).

Philosophical skepticism notwithstanding, the notion of personal moral responsibility remains (and should remain) relevant in bioethics as a field of applied ethics with a potential influence on public policy and concrete implications for resource allocation (Battin et al. 2009; Hansson 2022; Schmidt 2019; Brown and Savulescu 2019; Brown 2013; Verweij and Dawson 2019; Schwan 2021; Schmidt et al. 2016). Authors in bioethics have generally taken some version of a compatibilist approach, allowing for some personal responsibility in lieu of a consensus around the influencing power of social and political determinants of health. We are generally sympathetic to the view that individuals may have some responsibility in some cases for their health even though social determinants of health influence the extent of such responsibility as well the extent to which these individuals should be held accountable for the health outcome of their behavior (Schwan 2021). Such a view, however, further highlights the complexity in assigning individual responsibility and accountability, and seems to be in a minority in bioethics. We thus take perhaps a more fruitful path.

A recent account (Brown and Savulescu 2019) of responsibility attempts to evade the philosophical controversy over individual moral responsibility by considering the diachronic and dyadic nature of responsibility. ‘Diachronic’ means that “health-related behaviour is often the product of multiple choices and actions over time.” ‘Dyadic’ means that an agent’s health behavior is the product of both her and her close others’ actions. Unfortunately, as the authors themselves admit, such an expanded account may not apply to vaccinations which are ‘one-shot’ (or four-shot) behaviors. Their discussion (as well as others’ (Verweij and Dawson 2019)) is still helpful though as it suggests that the division of individual and societal responsibility is not as stark as it may appear. So while our experience with unvaccinated individual patients is the driving force for this paper, our focus is the reciprocal relations between clinicians and society (going some way perhaps towards a communitarian account of responsibility, which is the end-goal of the authors (Brown and Savulescu 2019)).

Another recent discussion (Brown et al. 2019) of responsibility argues that individuals merely have prudential rather than moral reasons to engage in healthy activities (or avoid unhealthy activities). This means that governments could and should assign responsibility as attributability rather than accountability (meaning, a higher and more demanding level of moral responsibility), and consequently engage in policies that facilitate healthy behaviors rather than punish individuals for unhealthy behaviors. This account further justifies the move from doctor-patient relations to doctor-society relations.

The questions most relevant here, then, pertain to the risks imposed upon HCPs caring for Covid-19 patients, and whether society has done enough to mitigate them. Actual risk, however, is not the only factor to consider. The way HCPs perceive risks has the potential to affect their lives and the lives of their loved ones and thus increase their burden. Below we thus consider whether a duty of care applies in the context of Covid-19 in light of both perceived and actual risks, specifically towards the unvaccinated.

## A duty of care during Covid-19

In caring for Covid patients, HCPs face different kinds of risks, including psychological risks and burdens and infectious risks (i.e. risk of contagion to oneself and others) (Upshur et al. 2005). In terms of psychological risks and burdens, HCPs worldwide have been facing increasing burn-out and professional fatigue because of the continuous need to wear Personal Protective Equipment (PPE) (Walton et al. 2020). Put simply, HCPs worldwide need to work much harder because of vaccine hesitancy.

Anecdotal evidence demonstrating the perceived and actual increased infectious risks for HCPs abound. We all surely had colleagues who are medical professionals and who were quarantined or sick with Covid-19. Images and stories of HCPs moving to separate apartments or living in tents will forever be imprinted unto our professional and societal memories (Bala 2020; Niehaus 2020). Families were then torn apart and separated because of the perceived risk and not solely the actual risk due to Covid-19.

Empirical evidence concurs: HCPs across the world are disproportionally affected by Covid-19. A systematic review and meta-analysis of nearly 12,000 HCPs worldwide found a 51.7% prevalence of Covid-19 infection (Gholami et al. 2021). A Somalian study not included in the review found the prevalence among HCPs to be 61% (Abdi et al. 2021). A large Scottish study that was not included in the review revealed increased rates of hospital admissions among HCPs and their families compared to the general population (Shah et al. 2020). Another study that similarly was not included in the review found that 39 (13.1%) out of 1497 personnel working at the largest hospital in Israel and who were vaccinated with the BNT162b2 messenger RNA vaccine (Pfizer–BioNTech) were positive for Covid-19. Out of these 39, 29 were HCPs. Eleven out of 37 personnel for whom data were available were infected by an unvaccinated patient or HCP; seven cases were specifically linked to a patient receiving non-invasive ventilation who was not known initially to be positive (Bergwerk et al. 2021). While comparisons with the general population are inherently problematic for various reasons, it seems reasonable to conclude that infection rates among HCPs are higher than the general population.

What explains the heightened risk to HCPs compared to the rest of the population? Limited research demonstrates that PPE is not fully protective against Covid-19 (Feldman et al. 2020; Verbeek et al. 2016), and this may partly explain such increased risk. Another reason—particularly relevant to emergency medicine—may be that asymptomatic infections often mean that patients are examined, discharged or admitted without any suspicion they are positive for Covid and contagious. In our respective institutions, Covid testing was not automatically done on all patients upon entry to the emergency department but only on those who fulfill certain criteria, namely respiratory symptoms and fever. Many patients with no fever or respiratory complaints ended up being tested positive for Covid after a routine PCR test prior to admission. While surgical masks—which at time of writing were mandatory in hospitals in Israel—reduce risk of contagion, patient contact, physical examination, and various procedures done by the un-suspecting emergency care provider plausibly increase the risk of contagion. Fortunately, vaccination of HCPs obviously decreases their risk of developing a major disease. The third reason for the increased risk—and the one we focus on here—is vaccine hesitancy.

Various vaccines against Covid-19 currently exist, and they have proven to be safe and effective, even in pregnancy (Wu et al. 2021; Lopez Bernal et al. 2021; Pouwels, et al. 2021). As of writing, many countries have introduced a third and even a fourth booster vaccine to further reduce the risk of severe disease, morbidity and contagiousness. The FDA currently approves such boosters for people in most age groups. These vaccines are currently offered for free, thus minimizing issues of national distributive justice.

An immediate question arising at this point pertains to vaccine effectiveness, e.g. effective in what? The literature is fairly conclusive in demonstrating that the BNT162b2, CoronaVac, ChAdOx1-S, and mRNA-1273 are effective in reducing mortality and severe disease/hospitalization due to all covid-19 variants and subvariants, including Omicron B.1.1.529 (McMenamin et al. 2022; Andrews et al. 2022). This effectiveness positively correlates with the number of vaccine doses. Vaccines are also effective against mild disease but such immunity wanes within several months (Andrews et al. 2022). Vaccine effectiveness in reducing the risk for severe or mild disease wanes faster among elderly individuals compared with younger individuals (Braeye et al. 2023). The vaccines BNT162b2, mRNA1273, Ad26.COV2.S, ChAdOx1 were also proven to reduce transmission rates of Covid variants, including the Omicron variant (Braeye et al. 2023; Harris et al. 2021; Lyngse et al. 2022). Such lower transmission stems either from reduced infectiousness of the infected individual and/or susceptibility of the vaccinated individual. Thus, vaccinated people tend to develop severe disease less often compared with unvaccinated people, and thus pose less burden on the healthcare system and HCPs. Vaccinated people also tend to be less contagious than nonvaccinated people.

What does this all mean regarding the direct Covid risk towards HCPs? Vaccinated HCPs are less likely to suffer severe disease by all Covid variants, thus weakening the normative argument elaborated below. Note however that the known empirical data on the Omicron variant adds an extra complexity for the normative argument elaborated below. Omicron in general tends to cause less severe disease. At the same time however, the immunity against it conferred by vaccination is more short-lived compared to other variants. Thus, even HCPs who are vaccinated are at increased risk of developing at least a mild disease and subsequently transmit the disease to their surroundings.

Yet, vaccine hesitancy persists worldwide (Machingaidze and Wiysonge 2021). As this paper was being written only 46% of Australians have received at least one vaccine. In New Zealand only 38% have received at least one vaccine.^5^ In these rich countries, vaccine hesitancy mostly accounts for such low rates of vaccination. The US is heterogenous as usual: while vaccine hesitancy at time of writing was estimated to be 3.8% in the San Francisco County, California, it was estimated to be 26% in Garfield County, Montana.^6^ Only 65% of people aged 80 and above in China have received a booster.^7^ A survey conducted among 23,000 people in 23 different countries in June–July 2022 found that only 79.1% accepted vaccination (Lazarus et al. 2023). Only 86% among 4071 respondents in Saudi Arabia received vaccination in June-July 2021, with women being less likely to accept vaccination and only 42% of all respondents willing to vaccinate their teenage children (AlJamaan et al. 2022).

In light of these data and the preceding discussion, a duty of care towards the unvaccinated must be contested. Authors writing in the context of Covid-19 generally argued for a duty of care, again under a condition of societal reciprocity and protection (Johnson and Butcher 2020; Bakewell et al. 2020). But they have not considered a duty of care specifically towards the unvaccinated—we turn to that next. Beforehand, however, one argument should be discarded.

As the quote from the AMA above, authors have argued that HCPs should protect themselves to assure that they could care for other current or future patients (Bakewell et al. 2020). A brief analysis though demonstrates that the validity of this argument is limited. First, there is no reason to worry about current patients who may be harmed because a HCP chose to treat a Covid patient without appropriate protective measures. SARS-CoV-2 requires several days of latency before causing symptoms or being capable of human-to-human transmission, so HCPs who are currently caring for patients are not at immediate risk of falling sick. Second, a concern for future patients is only justified in extremely strained medical settings where only one HCP provides care. In most clinical settings in high-income countries substitutes could be found, and patients would eventually be taken care of, albeit with some delay.

## A duty of care towards the unvaccinated

HCPs have a duty of care towards the unvaccinated the same way they do towards the vaccinated. To say otherwise would be a breach of justice—human beings deserve equal respect regardless of their geography, race, skin color, gender etc. Equal respect entails equitable access to health and healthcare such to allow for a fair distribution of opportunity (Just 2007). A duty of care, however, is a *prima facie* duty, which means it may be overridden by other considerations. One plausible consideration is the risk imposed upon HCPs and their families.

At least in some cases and to variable degrees, a failure to fulfill one’s moral responsibility should beget moral blameworthiness and accountability, defined as deserving to carry the burden of the outcome for which one is accountable (Schwan 2021). When people who are eligible and have access to vaccination refuse to receive vaccination, they unnecessarily risk infecting other community members, both in their close vicinity and globally. They also risk HCPs and their families. This is an unnecessary risk because of the proven necessity, effectiveness, and safety of vaccines in reducing both severe disease and infectiveness (i.e. one’s risks of becoming infected) (Eyre et al. 2022; Stokel-Walker 2022; Chin et al. 2022), and would remain so despite of any misinformation that has been spreading globally. Inasmuch people then may be held morally responsible for refusing vaccination, they should be held accountable for refusing vaccination. Imposing a burden on the unvaccinated then, may be justified in some cases. Pragmatically, however, identifying instances in which holding someone morally responsible and thus accountable is legitimate may prove wanting (Hansson 2022; Schwan 2021).

The unnecessary risk imposed on community members is morally significant because community members—including HCPs as community members—generally ought to care for one another or at least avoid unnecessarily harming one another.(Battin et al. 2009; Hansson 2022) This assertion may rely on any number of existing egalitarian accounts, seeking to ensure equal capabilities (Nussbaum 2007), liberties and opportunities (Rawls 1971).

In addition, the unnecessary risk imposed upon HCPs takes on an even greater moral significance because HCPs, who possess a special positive duty, have a reciprocal right to be cared for. Since such a right to be cared for is a necessary condition for upholding a special positive duty, HCPs do not hold such a duty when some threshold of the quantity of unvaccinated people in a community is reached. In light of such a right to be cared for, and in order to again validate a duty of care between HCPs and Covid patients (or those suspected of having Covid—again, particularly relevant to emergency medicine physicians), patients ought to receive vaccination. Until then, HCPs’ obligation to care for these patients is *supererogatory*.

The right of HCPs to be cared for thus plays an important normative role in the argument, and should therefore be justified. A deeper analysis of the right to be cared for is also necessary to explain the seemingly arbitrary focus on HCPs as moral agents entitled to be the beneficiaries of a right to be cared for rather than other moral agents who are not HCPs.

## A right to be cared for

One way to justify or ground a right to be cared for is to refer to the principle of reciprocity, espoused, as mentioned, by virtually all relevant authors and reports. This principle seems intuitive enough to be accepted by all sides of a social contract and indeed plays into the structure of a social contract. Such a social contract argument was used by classical and modern scholars to devise and legitimize forms of government or public policies. Reasonable people (or people who can engage in public reason) who plausibly represent present and future generations, and the government are commonly considered the main stakeholders.

The concept of the social contract has been employed in the medical context, denoting a pact between society, individual patients or potential patients, government, the biomedical institution and individual practitioners, and healthcare administrators (Cruess and Cruess 2008; Cruess 2006; Rosen and Dewar 2004; Bhugra 2014). As part of such pact, society may and should have certain expectations from HCPs, generally expressed as medical professionalism, and HCPs in turn may and should have certain expectations from society, particularly the expectation to optimize conditions for the administration of care: staff, equipment etc.

In this paper, the social contract between HCPs and patients or that between HCPs and society is extended (or rather simplified) to a social contract that is closer to its historical and theoretical origins compared to the one described above. It is a social contract between society, as a collaborative union of individuals, and patients or potential patients. On one metaphorical side stand members of the community who are capable or potentially capable of contributing financially and otherwise to the communal healthcare system. On the other metaphorical side stand patients or potential patients. The healthcare system ought to treat all members fairly and adequately. Fair and adequate care includes access to adequate healthcare, which in itself includes access to HCPs who are able to provide proper care in a timely manner. HCPs who must work under unreasonable conditions and excessive or unnecessary risks can hardly perform properly. Further, they are plausibly justified in demanding reciprocity: they bear greater risks and burden compared with other community members and should thus receive some benefits in return. HCPs then can claim a right to be cared for as part of a wider social contract between society and patients or potential patients (Reid 2005). If HCPs are not cared for, they will not be able to provide adequate care and patients will not have adequate access to healthcare. Society would then fail to respect the social contract.^8^ In addition, the reciprocity principle, potentially accepted by all parties to the social contract, would not be respected if HCPs are not cared for. Again, society would then fail to respect the social contract.

A right to be cared for may entail many components, including adequate pay, appropriate PPE, etc. A strong case has been made that different components may apply differently both qualitatively and quantitatively based on the circumstances, and that HCPs could respond in a task-specific manner (Johnson and Butcher 2021). In other words, a right to be cared for includes access to N-95 masks only when a HCP is asked to care for a patient with an airborne disease; only in these circumstances can a HCP be said to hold strictly a supererogatory duty of care. Our argument here then may be perceived as concentrating on one such component in the very specific case of Covid-19 at the present time: effective vaccination against existing Covid-19 variants. If a future variant is discovered to be immune to vaccines, then patients receiving vaccination would no longer be considered necessary to comply with the right of HCPs to be cared for.

One counter-argument may be comparing vaccine hesitancy to smoking or obesity, which pose a significant extra burden on healthcare systems globally—if HCPs do not owe a duty of care towards the unvaccinated, does it mean that they also do not owe such a duty towards people who choose to smoke or to be obese? This argument misses the mark. The main point to consider is the risk to HCPs and by proxy to their families. The main point is not the increased burden the unvaccinated pose on the healthcare system. While second-hand smoking may pose some extra-risks to HCPs, it is not as significant as the risk of being infected with Covid and subsequently spreading the virus to one’s family.

Another counter-argument involves other activities or omission of self-care that potentially put individuals at increased risk—are HCPs obligated to care for HIV/AIDS patients who were infected because they refused to use protective measures during sexual encounters for example? As mentioned above, risk to HCPs is one key factor in deciding whether a duty of care exists, and above we saw that the risk of contracting HIV was too small to obviate a duty of care. In other cases, where risk is more significant, our conclusion may certainly apply. The same goes for burden—HCPs may not have a duty of care towards patients whose behavior unnecessarily^9^ strains the healthcare system and significantly burdens HCPs.

Another important point to remember is that while individuals indeed have a reciprocal duty to receive vaccination, societal obligations do not end there. Rather, national governing bodies have a reciprocal duty to maximize vaccination rates by implementing evidence-based campaigns and interventions that follow well-accepted principles of public health ethics, such as transparency and fairness (Childress et al. 2002). This duty again stems from a wider social contract between society and patients.

The last point flushes out further commentary, distilling the discussion around personal responsibility above. Refusal of those eligible to be vaccinated to receive vaccination often stems from a deep mistrust in the system or limited access to reliable sources of information. These in turn may stem from deep historical and present disadvantages and structural injustices (WHO 2014). Vaccine hesitancy in such contexts is at least understandable, and addressing it requires cultural and ethical sensitivity. This is the reason the argument here is mostly directed at the level of local, national, and international communities rather than individuals—practically, efforts should focus on addressing structural reasons for vaccine hesitancy rather than sanctioning recalcitrant individuals. This paper should not be understood as arguing that the unvaccinated should be punished. It should also not be understood as arguing that HCPs should not care for the unvaccinated. Rather, it should be understood as arguing that HCPs should be particularly praised and reciprocated because they are not ethically obligated to care for the unvaccinated—they do it for supererogatory reasons. Regardless of vaccination status, when patients call—they come.

Also from a practical perspective, one difficulty will be to discern vaccinated from unvaccinated patients—how would we know? Patients may simply lie or refuse to disclose (and both authors have experienced many such instances). There does not seem to be any satisfying solution here, other than mostly relying on patients’ statements. Requiring patients to show proof of vaccination or so-called vaccination passports (Jecker 2022) is of course a potential solution, but it may engender injustices as some patients simply do not own smartphones or printers to show such proofs. Paper certificates also come in various forms worldwide and are easy to fake. Additionally, asking patients to provide proof of vaccination prior to treatment may dampen the patient-doctor relationship and lead patients to think that receiving medical care hinges on their vaccination. That is not what we are advocating here—unvaccinated patients should receive care; we just need to realize that HCPs are acting beyond their call of duty in providing that care.

A bit more nuance is needed to distinguish national and international communities, as the obligations of individuals and communities towards HCPs vary both in nature and degree depending on their geographical location. A person who refuses vaccination in Africa poses very little risk to the HCP working in North America—perhaps that of increasing the risk for the emergence of new variants—compared to a non-compliant person attending the hospital in which the HCP works. This and other morally relevant factors should be considered when calculating the scope of duties and corresponding rights of HCPs. In our context, one may wonder then about the threshold of vaccination rates in a given local, national, or international community to fulfill the appropriate communal obligations towards HCPs. Plausibly, if all eligible people in a given local community are vaccinated, a HCP’ duty to care for members of this community holds even in light of no compliance in a community on a different continent. Conversely, if HCPs have any duty of care towards people in other continents (which is highly controversial, although the present authors think they do), such duty is plausibly reduced in non-compliant communities. Delving into such nuance however will led us too far astray, as our goal is to articulate and conceptually justify a general right of HCPs to be cared for in the context of Covid-19, with one concrete specification—vaccination. Pragmatically, our discussion may imply that a HCP strictly holds a supererogatory duty to treat the individual unvaccinated patient standing in front of the HCP.

## Concluding remarks

HCPs, emergency medicine practitioners included, have a duty to care for all patients as best as they can. That duty is not absolute however, and whether HCPs ought to discharge it depends on several variables, primarily their competence and risks involved. HCPs also have a reciprocating right to be cared for, which means that society has an obligation to reciprocate a duty of care by minimizing the risks faced by HCPs and perhaps also by remunerating them. Reciprocity in this case entails maximizing vaccination rates.

Authors have lamented the lack of attention to the duty of care in clinical professional guidelines (Malm et al. 2008; Upshur et al. 2005), apart from the AMA code of ethics mentioned above (Ruderman et al. 2006), some limited and general UK guidelines (Johnson and Butcher 2020), and Canadian emergency medicine guidelines (Bakewell et al. 2020). Unfortunately, American (and Israeli) emergency medicine guidelines do not specifically address the duty of care as well, thus leaving individual clinicians or groups to their own devices. This paper would hopefully galvanize and inform the creation of a specific relevant national or international guidance, perhaps particularly pertaining to emergency medicine practitioners.

## Footnotes

1 Vaccine inequity and hesitancy has made the Omicron variant more likely, scientists say | CNN accessed 8/2023.

2 U.S. COVID-19 vaccine tracker: See your state’s progress—Mayo Clinic accessed 10/21.

3 Supererogation (Stanford Encyclopedia of Philosophy) accessed 11/2022. By claiming that supererogatory acts are praiseworthy by default we adopt the most common view in the literature. Not all authors agree however (Archer 2016). For an account of moral supererogation that does necessitate costs, see Benn (2018). Benn argues that the optionality character of supererogatory acts should be understood along the lines of comparative costs: if an agent does the bare minimum to fulfill a duty her act would not be considered optional and thus would not be considered supererogatory. If an agent goes beyond the bare minimum in fulfilling a duty, then her action is supererogatory.

4 An additional question revolves around the nature of care a clinician has a duty to provide and whether it may include palliative as well as curative care (Holt 2008).

5 Coronavirus (COVID-19) Vaccinations—Statistics and Research—Our World in Data.

6 Vaccine Hesitancy for COVID-19 | Data | Centers for Disease Control and Prevention (cdc.gov).

7 China touts vaccination progress as it looks for a path to reopening (cnbc.com) accessed 12/2022.

8 Obviously, the same deliberation plausibly defends a right to be cared for owed to other, so-called ‘essential’ professionals.

9 Defining what is necessary and what is unnecessary is of course tricky, but will not be further discussed here. In any case the discussion here would track that revolving around personal moral responsibility.
